# Spatiotemporal predictions guide attention throughout the adult lifespan

**DOI:** 10.1038/s41539-024-00281-3

**Published:** 2024-11-21

**Authors:** Nir Shalev, Sage Boettcher, Anna C. Nobre

**Affiliations:** 1 Department of Gerontology, University of Haifa, Haifa, Israel; 2 The Institute of Information Processing and Decision Making (IIPDM), Haifa, Israel; 3 Department of Experimental Psychology, University of Oxford, Oxford, UK; 4 Oxford Centre for Human Brain Activity, Wellcome Centre for Integrative Neuroimaging, Department of Psychiatry, University of Oxford, Oxford, UK; 5 Wu Tsai Institute, Yale University, New Haven, CT USA

**Keywords:** Human behaviour, Cognitive ageing

## Abstract

Older adults struggle with tasks requiring selective attention amidst distractions. Experimental observations about age-related decline have relied on visual search designs using static displays. However, natural environments often embed dynamic structures that afford proactive anticipation of task-relevant information. We investigate the capacity to benefit from spatiotemporal predictions across the adult lifespan. Participants (*N* = 300, aged 20–80) searched for multiple targets that faded in and out of displays among distractors. Half of the targets appeared at a fixed time and approximate location, whereas others appeared unpredictably. Overall search performance was reduced with age. Nevertheless, prediction-led behaviour, reflected in a higher detection of predictable targets, remained resistant to aging. Predictions were most pronounced when targets appeared in quick succession. When evaluating response speed, predictions were also significant but reduced with progressing age. While our findings confirm an age-related decline, we identified clear indications for proactive attentional guidance throughout adulthood.

## Introduction

Attending to goal-relevant information while ignoring distractions is necessary for adaptive behaviour throughout our adult life. When cycling on the road, we are on the lookout for potential hazards coming in and out of sight. To navigate successfully, we must prioritise relevant signals over irrelevant information. Such a prioritisation process is often simulated in the laboratory using visual search tasks, in which participants are tasked with finding a target among distractors^1^.

Traditionally, work on attentional guidance in visual search has focused on the interplay between top-down attentional biases towards task-relevant features (e.g., while searching for my mobile phone, I may attend to someone else’s phone^2^) and bottom-up biases towards salient information (such as noticing a salient signpost or advertisement^3^). Attention is also guided by our prior experiences shaping our anticipation of the location and attributes of stimuli^4–7^.

Throughout our lifespan, the cognitive system continuously changes and, consequently, so does search performance. Visual search studies reveal a characteristic performance decline with ageing between early and older adulthood (e.g. refs. ^8–11^,). However, as the control of spatial attention is driven by a complex constellation of interacting processes, it is unclear what underlies the decline. Theoretical accounts present evidence for an age-related reduction in multiple aspects of cognition, all relevant to visual search. For example, one dominant account emphasises a reduction in top-down control^12–14^. According to this view, ageing is associated with increased sensitivity to distracting, task-irrelevant information^11,15–17^. An alternative account considers the role of perceptual decline as a leading cause for reduced performance (e.g. refs. ^18,19^,). Other researchers have highlighted the slowing in overall visual processing speed (i.e., the processing rate of visual items) as a leading cause of poor search performance^20,21^.

An additional factor for consideration of search performance in ageing is the influence of memory. In contrast to the ample evidence showing how search is impaired with age, evidence also suggests that reliance on prior experience in search can be preserved. Some studies have shown that older adults are comparable to younger adults in using probabilistic information about target location and features to improve performance in a visual search task^22,23^. A different line of work showed a similar capacity of younger and older adults to learn and utilise associations between targets and display configurations in visual search (“contextual cueing”)^24–26^. Additionally, older adults have been shown to rely on expectations about the location of search items in realistic scenes based on contextual information and semantic knowledge^27–30^. Younger and older adults are also similar in their overall accuracy when memorising a set of visual items and then searching for them among arrays of random objects (“hybrid search”)^31^.

The evidence showing that experience-driven attentional guidance in older age remains intact has important consequences. Real-life search tasks are temporally extended and dynamic, and prior experience plays a significant role in determining where and when attention should be allocated. A daily task, such as crossing a street, elapses over time with continuously changing stimulation. Relevant and irrelevant items appear and disappear from view. To achieve our goals, we must focus and gather information across the duration of our task. It is not enough to find the first car, but rather we must monitor the traffic continuously to cross the road safely. However, to date, most of the research on attentional guidance through the lifespan relies on tasks using static stimulus displays.

Recently, we introduced a new experimental design to investigate temporally extended visual search in dynamic contexts and the role that spatiotemporal predictions play^32^. Our task simulates the temporal dynamics of real-life search: Participants search for multiple targets that gradually appear and disappear during extended trials. Critically, some targets appear predictably in their timing and approximate location, and we test whether such regularities benefit behaviour. We discovered that both young adults and children form and utilise spatiotemporal predictions to guide attention during dynamic search tasks^32,33^. However, it is unclear whether such a pattern is preserved throughout the adult lifespan and with ageing.

Previous studies have shown that knowing the location and identity of targets leads to performance benefits throughout adulthood^22,23^. Identifying such preserved abilities in older age is important, given the overall lower abilities when performing a naïve search^11^. Furthermore, using prior experience is a more ecologically valid approach to studying search, as in most real-life scenarios we have some knowledge about the whereabouts of things. When considering the dynamic properties of natural environments, an important question is whether prior knowledge about the timing of targets is also meaningful. Limited evidence in the temporal literature suggests that it is possible, although the evidence is conflicting and gathered in a relatively impoverished context. Some studies have compared younger and older adults in tasks requiring the detection of a single target that appeared predictably or unpredictably and have shown comparable benefits for both age groups^34,35^. However, other studies have found impaired^36^ and attenuated^37^ benefits as a function of ageing. This body of work provides insights into fundamental timing mechanisms over the adult lifespan, using simple stimulus arrays and minimizing spatial factors. In our research, we will explore temporal predictability in a perceptually challenging and dynamic search task.

The current study investigates how people in early to older adulthood utilise temporal predictions during a dynamic and continuous search task. The unique task design requires participants to extract complex spatiotemporal regularities from a constantly changing display, while concurrently facing visual distractions. The regularities can then be used to anticipate and guide attention. Three hundred participants between 20 and 80 years old searched for multiple targets appearing and disappearing among distractors in a dynamic search display. The spatiotemporal predictability of targets was manipulated within trials to measure the capacity to learn and use regularities to guide attention. We also estimated the contribution of temporal preparation driven by the mounting probability of targets appearing over time.

## Results

We present the primary analysis of performance, with a focus on target predictability, response-onset interval, and age as the main predictors.

Analysis of the hit rate for finding targets in the dynamic visual-search task revealed main effects of Predictability (*β* = 0.22; SE = 0.06; z = 3.36; *p* < 0.001), Response-Onset Interval (*β* = 0.12; SE = 0.03; z = 3.7; *p* < 0.001), and Age (*β* = −0.02; SE = 0.002; z = −8.82; *p* < 0.001), alongside a two-way interaction of Interval and Predictability (*β* = −0.01; SE = 0.03; z = −2.9; *p* = 0.003). Age did not interact with Predictability (*p* = 0.14) or Response-Target Interval (*p* = 0.67). The three-way interaction among all factors was also not significant (*p* = 0.058).

**Fig. 1:**
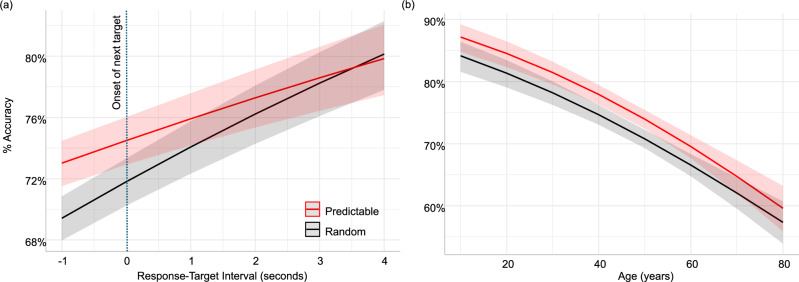
Predicted accuracy based on the fitted generalised linear mixed-effects model. The plot was generated using the ‘ggeffects’ package in R^38^ based on the output of the statistical model used for analysing ‘hit rate’ as a function of Age, Responses-Onset Interval, and target Predictability. **a** The x-axis represents the passage of time between the current target and the response to a previous one. In cases where the last target was omitted, we calculated the time between the onset and the previous response that was made. In this figure we focus on the predicted accuracy following in range between −1 second (meaning the response to target N-1 was made after the onset of target N) and 4 s (representing the longest possible time between sequential targets). The dashed line marks the onset of target N. The red line represents the detection of predictable targets, and the grey line represents the detection of variable targets (with confidence intervals). **b** Illustration of the main effects of predictability and age, showing that prediction benefitted behaviour irrespective of age.

The main effects indicated that hit rates for targets improve with predictability and longer intervals, while they decrease with age. We visualized the data to guide the interpretation of the two-way interaction. We calculated the model-based estimated marginal means for the Predictability and Interval factors using the ‘ggpredict’ package in R^38^, and plotted these model-based estimated values of accuracy. The observed pattern (Fig. 1) helps reveal the source of the interaction. This pattern was driven by the increasing influence of predictability on performance when responses to targets were in close proximity to the onset of the next target. For the longest intervals, performance was highest, and the predictability status did not influence behaviour. However, when responses were closer to or exceeded the onset of the next target, observers of all ages were more likely to select a predictable target.

Analysis of the reaction times in the dynamic visual-search task revealed main effects of Predictability (*β* = 0.015; SE = 0.006; t(270.5) = −2.239; *p* = 0.025), Response-Onset Interval (*β* = −0.14; SE = 0.007; t(442.4) = 18.684; *p* < 0.001), and Age (*β* = 0.15; SE = 0.02; t(294.2) = 7.972; *p* < 0.001), alongside a two-way interaction of Response-Onset Interval and Age (*β* = 0.02; SE = 0.007; t(440.5) = 2.788; *p* = 0.005). All other effects were not significant (all *p*’s > 0.063).

**Fig. 2:**
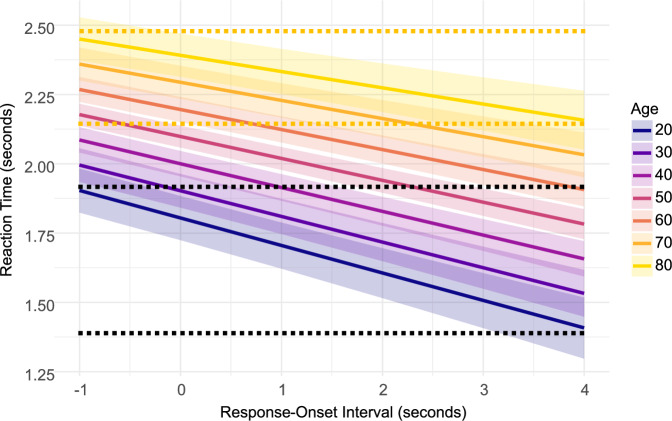
Predicted reaction times based on the fitted linear mixed-effects model. The plot was generated using the ‘ggeffects’ package in R^38^ based on the output of the statistical model used for analysing ‘reaction time’ as a function of Age, Responses-Onset Interval, and target Predictability. We plotted the model-based estimated marginal means for the Response-Onset Interval factors at seven representative ages: 20, 30, 40, 50, 60, 70, and 80. The Y-axis represents the reaction times in seconds. The X-axis represents the Response-Onset Interval in seconds. For illustration purposes, the dotted line represents the upper and lower bounds of the youngest age (20, marked in dark blue) and the oldest age (80, marked in yellow). The illustration highlights the source of the interaction between Age and Response-Onset Interval, with a larger change in reaction times with declining age.

Reaction times were faster for predictable targets. In addition, reaction times improved overall with longer Response Onset Interval and slowed with advancing age. To help guide the interpretation of the interaction we plotted the data in Fig. 2. As seen in the figure, the benefit in reaction times as a function of the Response-Interval Onset decreased with age.

## Discussion

Overall search performance declines with age: in our sample, age was negatively associated with accuracy and positively associated with response times (i.e., slower responses). However, despite advancing age, older participants were capable of benefitting from spatiotemporal predictions in extended dynamic search although the effects also diminished systematically over the lifespan. Accuracy benefits were dependent on shorter target-onset intervals, and significant response speeding also diminished as RTs became slower overall. The findings open many doors for further exploration of cognitive limitations in older age alongside preserved abilities.

Our results accord with the idea that experience and learning continue to play a crucial role in visual search throughout adulthood. Evidence, including our own data, has accumulated to propose a more nuanced perspective on attention control as people age in comparison to traditional views. Although there may be age-related declines in cognitive control^12,39^ and sensory processing^18,19^ that reduce both top-down and bottom-up signalling, memory-driven control appears to consistently compensate for these deficits. Long-term semantic knowledge helps guide attention in realistic scenes between younger and older adulthood^27,28,30^. In more immediate contexts, learning from environment patterns, as demonstrated in contextual cueing tasks (Howard et al.^24^; Jiang et al.^25^; Merrill et al.^26^), shows remarkable resilience to aging. Likewise, participants of various age groups benefit from having prior knowledge about the features and locations of targets they search for^9,22,23^. Our data also align with previous studies indicating preserved temporal anticipatory behaviour in older adults^34,35^. However, to our knowledge, this is the first study to examine the combined effects of spatial and temporal regularities on performance in dynamic contexts across the adult lifespan.

Our findings may be surprising given some broad assumptions about poor search performance in the ageing population. Individuals up to 80 years of age extracted highly complex task-relevant regularities embedded within perceptually loaded displays, even if mean performance was reduced. Accordingly, we found evidence for learning of regularities even when detecting fewer targets, highlighting a potential dissociation between learning-based guidance and the ability to overcome competition when searching in dynamic contexts.

Two kinds of temporal structures contributed to performance in the task: target predictability (i.e., learning the exact timing of predictable targets) and response-onset interval, which reflects the timing between sequential responses on behaviour. When evaluating the contribution of the response-onset interval to performance, we identified an interaction with target predictability. Longer intervals were associated with a reduction in reliance on predictions alongside improved performance. This pattern may have been driven by a ceiling effect, whereby individuals reached their peak performance when they had more time to search for and prepare for the next target to appear. Another perspective on these data is that in cases where the task was more demanding—i.e., when participants responded to one target in proximity to the next one—they adaptively resorted to relying on predictions

When evaluating reaction times as a dependent variable, across all age groups, participants were overall faster when they had a longer anticipation time. This is in line with a substantial body of literature on the behavioural benefits of temporal preparation (e.g. refs. ^40,41^,). We also identified a two-way interaction between age and interval: for older adults, the benefits of the conditional probability were attenuated in comparison to younger adults. Nevertheless, we have seen a similar trend across all ages, which only differed in its magnitude. The findings may reflect age-related differences in closely related mechanisms, such as slowing of processing speed^20^ or a reduction in alertness^42^, leading to noisier performance. Another explanation may be related to slower disengagement of attention from visual targets, which may increase interference in sequential behaviour in the context of the Dynamic Visual Search^43,44^. However, we suggest interpreting these findings with caution. A combination of overall lower accuracy for older people and the fact that participants were not instructed to respond quickly can explain some of the variability.

More broadly, our study presents an interesting case for the feasibility of conducting online research with older adults. In recent years, there has been a significant increase in the use of online cognitive studies, which have produced highly reliable data^45^. However, research involving older adults is more limited, potentially due to challenges in accurately assessing cognitive decline remotely. Although we acknowledge the limitation of not controlling for cognitive issues, we were still able to demonstrate similar learning effects across all age groups. Future research could enhance our findings by measuring additional cognitive dimensions and exploring individual differences.

An exciting future direction would be to integrate spatiotemporal regularities into dynamic, realistic search scenes. Such a design would not only enhance ecological validity but also enable exploration of whether spatiotemporal regularities guide attention across adulthood based on long-term expectations. In our study, we utilized a well-controlled artificial environment to maximize the effects of trial-by-trial learning of regularities. However, in real-life scenarios, some spatiotemporal regularities are likely stored in long-term memory, such as our expectations regarding the timing of traffic lights or the speed of an approaching car. Experience with realistic stimuli has been shown to be a meaningful source of attentional guidance^27,28,30^. To the best of our knowledge, however, the influence of prior experience with spatiotemporal regularities has not been tested with dynamic objects and scenes.

Our findings present an exciting picture of attentional guidance in older adults. While many studies have emphasized the substantial decline in attention control over the lifespan, our study highlights a remarkable sparing of a significant factor contributing to goal-directed behaviour. Extracting task-relevant regularities while facing distraction and using those regularities to optimize performance is a fundamental aspect of how attention is controlled in our everyday lives. Future studies can further explore whether temporal and spatial predictions can independently enhance behaviour in a continuous search task and whether any interaction between the two is influenced by age differences. The importance of our work lies in demonstrating how these two sources combine. To our knowledge, this is the first study to show how proactive spatial behaviour is guided by learning and representing the timing of events in both young and older adults. When considering all factors, we identify abilities that decline (overall search) versus those that are maintained (predictions) and can be adaptively used for compensation.

## Methods

All experimental procedures and protocols were reviewed and approved by the University of Oxford Central University Research Ethics Committee (CUREC Ethics Approval Reference: R74132/RE001).

A sample size of 300 participants between the ages of 20 and 80 was based on previous studies using equivalent experimental tasks. Our previous findings with younger adults, which we replicated in multiple studies (e.g. refs. ^32,33,46,47^,), indicated that a sample size of 25 participants yielded strong statistical power greater than 0.9 to observe performance benefits of spatiotemporal predictions. Because we considered that effect sizes could diminish with age, we selected this high level of power and recruited 25 participants within twelve equally sized “age brackets” between 20 and 80 years old. As a result, our sample contained twelve equal groups of 25 participants distributed between the ages of 20 and 80 years.

We used Prolific, a participant recruiting platform for online experiments^48^. Inclusion criteria involved having normal or corrected-to-normal vision and no diagnosed psychiatric or neurological condition. Participants were required to have completed at least ten previous studies on Prolific and achieved an approval rating of 80% or more (percentage of studies on Prolific for which each participant’s data had been approved). All participants used a personal computer to complete the study (i.e., the experiment did not support phones or tablets).

We recruited 329 participants to reach 300 data sets of sufficient quality for analysis. Data from twenty-nine participants did not reach the inclusion criteria (see below) and were replaced. The resulting cohort (132 male, 168 female) had a mean age of 50.5 years *(SD* = 17.3 yr; range 20–80). Twenty-nine individuals were left-handed, and the rest were right-handed (based on self-report). Participants provided informed consent and were compensated for their time at £8 per hour. The experimental task was generated using PsychoPy^49^ and was hosted on Pavlovia (http://pavlovia.org). Briefings were carried out using Qualtrics (http://qualtrics.com). The experimental code is available at OSF (DOI: 10.17605/OSF.IO/8UPY5).

**Fig. 3:**
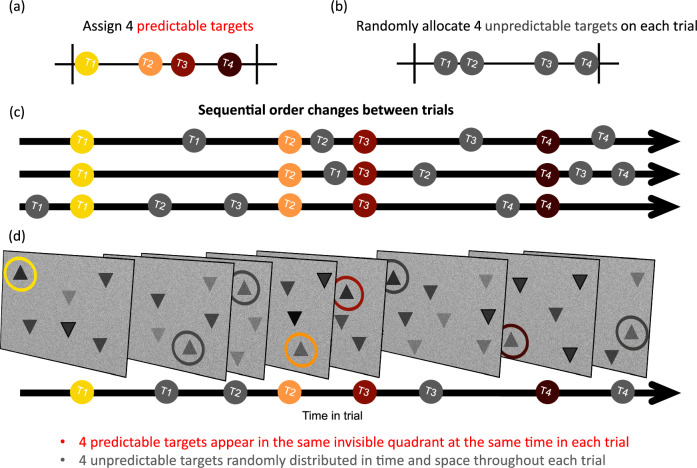
Illustration of the dynamic search task. **a** Each trial was divided into four time bins. Four predictable targets appeared in each trial, and each target was pre-assigned to each one of the four bins. **b** The other four unpredictable targets were assigned randomly on each trial, one target to each of the four bins. **c** The coloured circles represent predictable targets, which always occurred at the same time-point within their assigned bin across a block of trials. The grey circles represent the four random targets, which were randomly distributed over time. Three examples are provided showing that the order of predictable and random targets could vary across trials. **d** The four predictable targets were assigned to four different quadrants, which were kept constant throughout a block of trials. The quadrants in which random targets appeared were completely randomly determined. An example of a single experimental trial can be found on our OSF page (10.17605/OSF.IO/8UPY5).

Figure 3 shows the task. Participants were instructed to find eight upward-pointing triangles (‘targets’) on each trial and ignore downward-pointing triangles (‘distractors’). Trials lasted approximately fourteen seconds and consisted of eight targets and sixteen distractors fading in and out of the search display over its duration. The search display had a static white-noise background, and all target and distractor stimuli were black. Stimuli faded in and out of view slowly but did not move. The fade-in time was set to 1.33 s, gradually becoming visible until reaching a maximum of 80% opacity. The target remained on the screen for another 1.33 s and then faded out over another 1.33 s. Each stimulus was set to a size of .07 normalised units (length and width), with 1.0 being the full-screen size. Stimuli could appear in any location on the screen but not overlap with another. Whenever participants detected a target, they used their mouse or trackpad to click on it. A response was categorised as “correct” only if the participant clicked on the target while it was presented (i.e., within a time window of four seconds).

Of the eight target stimuli, four were spatially and temporally predictable (Predictable targets). On every trial, they appeared at the same temporal onset from the start of the trial and within the same quadrant of the full screen - top left, bottom left, top right, or bottom right. Location within the quadrant, however, remained variable. The onset times for predictable targets were pre-assigned as follows: we created twenty-four equally spaced time points between the earliest (0.6 s) and latest (10 s) possible onsets. The interval between the time points was approximately 0.41 s. The resulting linearly spaced segments were further split into four equal ‘bins’, each consisting of six equally spread onsets. To select the four predictable onsets for a given participant, one onset from each of the four time bins was drawn at the beginning of the experiment. Each of these four onsets was then randomly assigned to a different spatial quadrant. The onsets and their associated spatial quadrants then remained fixed for that participant throughout the experiment. Different participants had different intervals and interval-quadrant pairings for predictable targets.

The four remaining targets appeared at unpredictable quadrants and temporal onsets (Variable targets). Their timing was distributed pseudorandomly, by assigning each to a remaining, unassigned onset from the same uniform distribution irrespective of a time bin. To increase the overall temporal variability for stimulus appearance across trials, the exact onset times for each variable target were jittered further by adding or subtracting a value between 0 and 500 ms. These constraints ensured targets were roughly evenly distributed throughout the trial and avoided too many target events occurring at one time. All remaining sixteen onsets from the distribution we created that were not assigned to a target were used for distractors. The jittering approach was the same used for setting the timing of the targets. The locations of Variable targets and distractors were chosen randomly at the beginning of each trial and were unique (i.e., different stimuli never appeared in the same location within a trial).

Participants completed ten practice trials to become familiar with the task before completing a single block of 30 experimental trials, in which a total of 240 targets could be detected. During practice, all targets appeared at random times and locations (there were no ‘predictable’ targets). Each trial contained 8 targets and 16 distractors. In experimental trials, 4 targets appeared predictably and 4 appeared unpredictably. Participants were not informed about the predictability manipulation. Overall, there were 120 predictable and 120 variable targets per participant (excluding practice trials) and 480 distractors (all unpredictable). After each trial, observers received feedback indicating how many targets they found and how many trials remained until the end of the experiment. A new trial could either be initiated by the participant by pressing the ‘space’ key or start automatically after 5 s. In average, it took 12.6 min to complete the cognitive task (SD = 0.87 min; Min = 11.16 min; Max = 16.27 min).

Behavioural data were analysed using R (R Core Team, 2018). Inclusion criteria for data analysis required individuals to identify more than 33% of targets overall (across predictable and variable categories). The threshold was based on the detection of behavioural outliers with mean performance outside 1.5 times the interquartile range above the upper quartile and below the lower quartile. All the outliers identified performed below the lower threshold. Based on the exclusion criteria, we removed twenty-nine participants and recruited twenty-nine new participants of the same age to replace them.

Our primary dependent variables to evaluate prediction benefits for search performance in the dynamic visual-search task were hit rates and reaction times. Hit rates and reaction times have proved to be reliable and sensitive measures of the benefits of spatiotemporal predictability in young adults and children in our previous studies^32,33,47^. The data were analysed using mixed-effects modelling, followed by appropriate *post-hoc* analysis procedures. The hit rate was evaluated as a function of target predictability (predictable vs. variable) to test whether participants utilised regularities to improve performance. In addition, we modelled the passage of time between a response to a target and the onset of the next target (‘Response-Onset Interval’, or simply “Interval”). This variable captured changes in preparedness between targets. We focused on the timing between responses and subsequent target onsets to ensure we considered task events that influenced behavioural engagement with the task. Age (in years) constituted a between-participants factor to assess changes in utilisation of spatiotemporal predictions across the adult lifespan.

Responses to targets were classified as hits or misses and fitted using generalised linear mixed-effects models (GLMMs) with a binomial distribution. All responses were analysed. Data were modelled using three fixed effects: Target Predictability (Predictable vs Variable targets), Interval (continuous variable, ranging between zero and four seconds, for each of the Predictable and Variable targets; first targets were assigned their onset relative to the beginning of the experiment), and Age (continuous variable, between 20 to 80 in units of years). The analysis used the lme4 package (version 1.1-17^50^;. For the GLMMs, we report the regression coefficients β with the z statistic and use a two-tailed 5% error criterion for significance. The *p*-values for the binary accuracy variable are based on asymptotic Wald tests.

Response-Onset Interval and Age were scaled and mean-centred and entered the model as a continuous predictor. Modelling of the comparison between Predictable and Variable targets used a binary factor. The coefficients were represented by logits. Each model began with a maximal random-effects structure. This included intercepts for each participant, as well as by-participant slopes for the effects of Age, Interval, and Predictability. Full models such as these often lead to overparameterisation^50^. Therefore, we used a principal component analysis (PCA) of the random-effects variance-covariance estimates to identify overparameterisation for each fitted model^50^. However, in the current model, all the effects contributed significantly, and therefore the full model was retained for analysis of the Hit Rate. The GLMM random-effects structure contained the intercepts for each participant, as well as by-participant slopes for the effects of Predictability, Interval, and their interaction.

The same factors as in the Hit Rate analysis procedure were used to analyse reaction times: Predictability, Interval, and Age. We fitted the reaction times for correct target identification using a linear mixed-effects model (LMM). To remove extreme outliers, we converted all the reaction times to z-scores based on each participant’s mean and standard deviation. We filtered out responses that exceeded three standard deviations from the mean. In total, we removed 340 observations (approximately 0.5% of the data). The resulting distribution was inspected visually using a q-q plot and compared to a distribution obtained from a log transformation to determine if a transformation was needed. Our raw data fit better with a normal distribution, so we used the raw data for the statistical analysis.

We used the same factor and model structure as the GLMM used to analyse the accuracy data. For the LMM, we report β with the t-statistic and apply a two-tailed criterion corresponding to a 5% error criterion for significance. The *p*-values were calculated with Satterthwaite’s degrees of freedom method using the lmerTest package (Version3.1-0^51^;). Response-target Interval and Age were mean-centred and entered the model as continuous predictors. Modelling of the comparison between predictable and variable targets used a binary factor. As with the accuracy data, we used a PCA of the random-effects variance-covariance estimates to identify overparameterisation for each fitted model and remove random slopes that did not contribute significantly to the goodness of fit in a likelihood ratio (LR) test^50^. For reaction-time analysis, the PCA approach indicated overparameterisation and supported a partial model. The LMM random-effects structure contained the intercepts for each participant and the by-participant slopes for the additive effects of Interval and Predictability only.

## Data Availability

The experimental code is available at OSF (10.17605/OSF.IO/8UPY5).

## Data availability

The data will be made available at OSF upon the acceptance of this paper.
